# 35 years of the *Brazilian Journal of Medical and Biological Research*

**DOI:** 10.1590/1414-431X20166153

**Published:** 2017-02-06

**Authors:** E.M. Rego, J. Pereira Leite, I. Bensenor, R. Chammas, J. Nogueira de Francischi, P.L. da Luz

**Affiliations:** 1 Departamento de Clínica Médica, Faculdade de Medicina de Ribeirão Preto, Universidade de São Paulo, Ribeirão Preto, SP, Brasil; 2 Departamento de Clínica Médica, Faculdade de Medicina, Universidade de São Paulo, São Paulo, SP, Brasil; 3 Instituto do Câncer do Estado de São Paulo, Faculdade de Medicina, Universidade de São Paulo, São Paulo, SP, Brasil; 4 Departamento de Farmacologia, Instituto de Ciências Biológicas, Universidade Federal de Minas Gerais, Belo Horizonte, MG, Brasil; 5 Instituto do Coração, Disciplina de Cardiologia, Faculdade de Medicina, Universidade de São Paulo, São Paulo, SP, Brasil

## Abstract

The authors pay homage to the three founders of the *Brazilian Journal of Medical and Biological Research* Profs. Lewis Joel Greene, Sérgio Henrique Ferreira and Eduardo Moacyr Krieger for their vision and commitment to divulge the scientific production of developing countries.

## Introduction

In 2016, the Brazilian Journal of Medical and Biological Research celebrated its 35th anniversary. During this period, the BJMBR published 49 volumes containing more than 6,000 peer-reviewed articles from authors working in the five continents. Through its policy of making the world's scientific and medical literature a public resource, the BJMBR became an important vehicle to divulge the scientific production of developing countries, whose quantity and quality are steadily increasing over time. The successful history of the journal is due mainly to the vision and commitment of its three founders, Profs. Lewis Joel Greene, Sérgio Henrique Ferreira and Eduardo Moacyr Krieger, to whom we pay homage in this issue. They transformed the *Revista Brasileira de Pesquisas Médicas e Biológicas* founded by Michel Jamra that was published in Portuguese into the *Brazilian Journal of Medical and Biological Research* published in English. The reader will have the opportunity to grasp the relevance of the work of these three leaders through the following articles written by Profs. Roger Chammas, Janetti Nogueira de Francischi and Protasio Lemos da Luz.

Establishing a scientific journal in a developing country such as Brazil in the early 1980's was a major challenge. There were many financial and political uncertainties and to keep the regularity and scientific rigor in manuscript analysis demanded the personal effort and long hours of dedication from the BJMBR founders. They championed for the development of science in low- and middle-income countries as a way to improve education (graduate and undergraduate), boost the economy and reduce social inequities. Moreover, they always stressed that the process was long and required resilience of the scientific community so that no corners would be cut and no compromise of the scientific rigor was allowed. The results go beyond the respect that the BJMBR gained among researchers, medical doctors, and health professionals.

Prof. Greene had a crucial role in the development of biochemistry in Brazil. He was one of the pioneers of analytical protein biochemistry and mass spectrometry and his studies on vasoactive peptides led to the characterization of the bradykinin potentiating factor, which was identified by Sérgio H. Ferreira. Unfortunately, this homage arrives late for Prof. Ferreira, who passed away in July 2016. He was one of the most successful Brazilian pharmacologists and his contributions not only to the understanding of the function of bradykinin, but also for the development of the angiotensin-converting enzyme inhibitors are internationally recognized. Prof. Krieger was one of the pioneers in translational medicine in Brazil, and his fundamental research contributed to better the understanding of treatment-resistant hypertension. Nevertheless, the common point among these three men is the outstanding capacity of mentorship, which may be witnessed by the successful careers of investigators trained in their laboratories.

The BJMBR is proud of being founded and nourished by such extraordinary individuals, and we are grateful to Drs. Chammas, Francischi and Lemos da Luz for their contribution.

**Figure:**
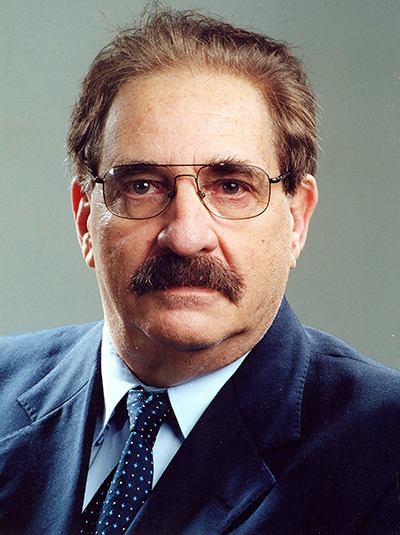


**Lewis Joel Greene**

## Scientists see no borders - a tribute to an honorary Brazilian

*By Roger Chammas, Instituto do Câncer do Estado de São Paulo, Faculdade de Medicina, Universidade de São Paulo, São Paulo, SP, Brasil*. rchammas@usp.br

It was at the Medical School at Ribeirão Preto, University of São Paulo, that the New Yorker Lewis Joel Greene (f1) rediscovered the collegial atmosphere of Amherst College and Rockefeller Institute, where he completed his graduate education in Biochemistry. Born to a family that valued culture and education, Greene completed high school at Peekskill Military Academy in 1951. Soon after, he entered Amherst College for Liberal Arts, aiming to prepare for Medical School after college. Both at Peekskill and Amherst, there was close contact of students with teachers, which largely promoted an academic environment favorable to learning and to the development of student skills and critical thinking.

At Amherst, academic research activities were part of the daily life of students, who followed mentors in the areas in the interface of Biology and Chemistry. Just before his last year at Amherst, Greene spent his vacation training in Richard Block’s laboratory at the Boyce Thompson Research Institute, Yonkers, New York. Block was famous for his analytical skills, and for creating innovative ways of separating biomolecules, such as amino acids and carbohydrates through paper chromatography, besides determining more and more sensitive ways to quantify these molecules. Despite the short time spent at Yonkers, Block influenced Greene who applied the principles of analytical biochemistry to a variety of scientific problems throughout his career. In his college thesis, supervised by Robert Whitney, Greene applied analytical biochemistry to understand which molecules would form upon ultraviolet irradiation (solar light) of simple molecules probably found in the origins of the Earth, such as nitrate and formaldehyde. The big question there was how essential amino acids were formed. The exercise of applying analytical tools to a still puzzling problem, and his performance during college, were recognized in his approval *cum laude* in the Honors Program of Amherst. Above all, his experience in research was decisive for the next steps of his career as an academician.

Greene was accepted at the Medical School of Rochester University; however, a timely indication for the then recently created graduate program at the Rockefeller Institute changed his mind. In 1955, Greene started his graduate studies in an experimental elite program at Rockefeller, where he stayed until 1962. The Rockefeller Institute in the 50’s and 60’s served as birthplace to Modern Cell Biology and Experimental Medicine. Indeed, two of the more prestigious journals in these areas, The Journal of Cell Biology and The Journal of Experimental Medicine had been edited by Rockefeller researchers and alumni for years. At Rockefeller, the commitment has always been with excellence - which started with the formulation of the questions to be solved scientifically. There, Greene trained with the biochemist C. Hirs and cell biologist G.E. Palade, studying the secretory process of the exocrine pancreas.

Greene worked out the conditions of cell fractionation and further characterization of the protein content of the subcellular fractions. Altogether, these studies led to the realization of transport between different intracellular compartments and the process of compartment-dependent zymogen activation. The application of electron microscopy techniques and the construction of the subcellular compartmentalization model gave George Emil Palade the Nobel Prize in Physiology and Medicine in 1974. The atmosphere at the Rockefeller Institute, where committed graduate students interacted with a large number of researchers such as Hirs and Palade, was again collegial. Students and researchers shared tables in the cafeteria daily, favoring the exchange of ideas turned into projects, transformed into papers, and promoting successful careers of Rockefeller’s alumni. There, Greene felt at home. After all, this was essentially the same atmosphere he had lived at Amherst.

Greene moved to the Brookhaven National Laboratory just after getting his PhD degree, in 1962, as an assistant biochemist in the Department of Biology, along with other colleagues and C. Hirs, his former PhD supervisor. In a few years, Greene was promoted to tenured scientist. However, at that time, the National Laboratory did not offer a graduate program for attracting PhD students, who were borrowed from other institutions, such as the Rockefeller Institute, where Greene still acted as an affiliate professor in Palade’s program. Brookhaven served as a hub for attraction of a number of visiting researchers from abroad, including several Brazilian researchers from the University of São Paulo and Escola Paulista de Medicina. Fellows of the Brazilian connection included the pharmacologists Maurício Rocha e Silva, Marina Lemos dos Reis, Sérgio Henrique Ferreira, Antonio Carlos Martins de Camargo and the biochemist Misako Sampaio.

The Brazilian connection at Brookhaven was first established by Maurício Rocha e Silva, who visited the National Laboratory. Greene mastered the language of protein chemistry, protein sequencing and amino acid analysis, which complemented fairly well the needs of the emerging generation of pharmacologists and biochemists of São Paulo at the end of the 60’s and early 70’s. First, there was an international agreement for technology transfer from Brookhaven to Ribeirão Preto. Five years later, after supervising at least five talented Brazilian scientists, it was time for Greene and his family to spend a sabbatical year in Ribeirão Preto. The sabbatical year of 1974 did not end. Greene and his family decided to stay in Brazil and chose Ribeirão Preto as their new home.

At the Medical School, Greene and his colleagues organized the first core facility of the University of São Paulo (an interdepartmental Center for Protein Chemistry), which served as a reference center for the entire country for years, besides training a large number of researchers who specialized in analytical protein biochemistry and in mass spectrometry for peptide analysis. Greene’s first contributions in Brazil were on vasoactive peptides, which led to the characterization of the bradykinin potentiating factor identified by Sérgio Ferreira as a specific peptide, allowing for its chemical synthesis. Then, Greene approached a variety of biomedical problems, including metabolism of peptide hormones, biochemical characterization of genetic disorders, nutrition, characterization of plant lectins, and diversity of hemoglobins. From protein chemistry and mass spectrometry, Greene’s interest moved into proteomics. The transfer of the Center for Protein Chemistry to the Hemocenter of Ribeirão Preto, and his affiliation to the Center of Cell-based Therapy allowed the consolidation of his research in proteomics in the context of cell biology, applied to fundamental problems such as stem cell biology, cancer biology and differentiation of hematopoetic cells.

Greene’s activities, however, extend much beyond his laboratory. In 1980, Greene opted for Brazilian citizenship and committed himself to make a difference in the promotion of Brazilian biomedical research. Together with Eduardo Moacyr Krieger and Sérgio Henrique Ferreira, Greene founded the Brazilian Journal of Medical and Biological Research, acting as its chief editor for 35 years. Palade used to call him ‘the honorary Brazilian’, and praised his commitment to the education of authors, reviewers and editors, all at different stages of our careers as researchers.

As an editor, however, Greene did not loose his collegial touch. The journal not only served as a repository of information or eventually as a forum for discussions, but it also served as the cafeteria table from the Rockefeller Institute, where authors met other researchers to improve their work, to have constructive criticisms, to improve their presentation logic, and to exchange ideas. The journal has been a school for many of us, either as authors, reviewers or editors. As his collaborator and coauthor, I learned to identify problems and to answer questions in an analytical way. As a reviewer, I learned to examine answers critically. As an editor, I learned to analyze the questions authors posed, and to value the educational role of an academic journal. In my opinion, altogether this is the legacy of the Brazilian Journal for my generation. This has been Greene’s silent scientific revolution for the country over the last 35 years.

**Figure:**
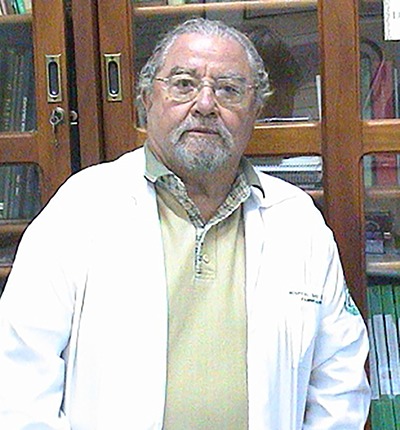


**Sérgio Henrique Ferreira**

## Tribute and testimony of a student

*By Janetti Nogueira de Francischi, Departamento de Farmacologia, Instituto de Ciencias Biológicas, Universidade Federal de Minas Gerais, Belo Horizonte, MG, Brasil*. janettif@icb.ufmg.br

These are my personal memories of a few aspects of the scientific relationship between a student and her supervisor, the late Professor Sérgio Henrique Ferreira (f2), one of the most successful Brazilian pharmacologists, who died on July 17, 2016. They refer to the years between 1977 and 1985, a relatively short period of Professor Sérgio Ferreira's long and productive academic life at the Faculty of Medicine of Ribeirão Preto (University of São Paulo). I also describe the academic environment in which many of the seminal papers published by S.H. Ferreira, particularly those on pain and inflammation, were written after his return to Brazil, during the same short period.

I remember saying “*This is what I want to do for the rest of my life!*” when I was accepted to work with Sérgio Henrique Ferreira at the Department of Pharmacology of the Faculty of Medicine of Ribeirão Preto, at the beginning of my scientific career at his laboratory. At that time, the beginning of 1977, he was already an internationally recognized Brazilian pharmacologist, chiefly for his earlier contribution to the field of anti-hypertensive drugs from his studies with *Bothrops jararaca* venom (1), that led to the ACE inhibitors.

At that time (1977), he had just returned from London, from a kind of “exile” from Brazil during the military dictatorship. There, he had worked with scientists who made the major advances in Pharmacology of the 70’s and 80’s, including Sir John Vane, who received the Nobel Prize for the discovery of the mechanism of action of aspirin-like drugs, Rod Flower, Mick Bakhle, Stuart Bunting, Gerry Higgs, Salvador Moncada, John Salmon, Brendan Whittle, and Tim Williams. These and many others I also had the privilege to meet and work with while a post-graduate student at Professor Sérgio’s laboratories or at the Wellcome Research Laboratories in Beckenham, England.

In the Pharmacology department in Ribeirão Preto, Sérgio frequently asked us students, named by the others in the laboratory as “the Sergettes” (Maria Salete de Abreu Castro, Berenice Borges Lorenzetti, Gloria Emilia Petto de Souza, and myself), if we had read this or that newly-published article in the inflammation field, or what did we think about the last pain paper in Nature or Science. (If I had not seen it, this question would shame me into running to the library to make good this omission!)

He spent most of the time in the Department writing his next manuscript. From time to time, however, he would appear at the student’s elbow, during an experiment to observe what was going on and tease the students with unexpected questions about the recently obtained results, showing a profound knowledge of the physiology and pharmacology of the research project.

During those years, he wrote crucial articles on peripheral pain, his main area of interest throughout his life, including the role of aspirin-like drugs and opioids (2,3), a pioneering concept that he put forward in the literature, which was followed by many other scientists in the world. Taking together the work of several of his students at this time, he was the first to put forward a role for macrophages as the body's alarm cells (4). Bearing in mind how little was then known or studied about the function of these cells, this paper was a remarkable prediction of what it is known now about these cells in the body’s response to inflammation and infection (5). On this occasion, we were trying to characterize pharmacologically a factor from lysed macrophages, in order to mimic what happens during an inflammatory reaction. When injected in rats, this factor induced a peculiar type of inflammatory pain response that, later on, was related to the activity of interleukin-1 (6,7). That correlation allowed Sérgio to initiate the concept of cytokines as major participants in the processes of inflammatory pain (7). The development of this concept has, more importantly, made significant contributions to our further understanding of pain in chronic inflammatory diseases. Other important research projects he developed in collaboration with students that did not attain the impact they deserved, in my opinion, included the studies related to “pain memory” (8) and what he called the “tele-antagonism” response (9).

He was a constant traveller. At this time, for us – who lived in a very conservative city in Brazil (Ribeirão Preto, São Paulo) – such travel, usually international, seemed spectacular, especially when he returned bringing new research tools and special supplies for the laboratory. Supplies that no one else could get at that time in the country were made available to answer the questions that had been raised before his departure. What an advance when he brought the first computer and we didn’t need to draw our graphs by hand anymore!

To question people fiercely was one of his ways to make Science; especially if his interlocutor was a man, as he was generally gallant with all the women who eventually surrounded him. Very often people would be upset by his way of questioning and, again, the Sergettes would step in to try to overcome the misunderstanding, but with variable results!

Brazilians are known by the international scientific community to have a peculiar use of the English language, which added, to some extent, to their difficulties in (English) listening and writing. Sérgio tried to compensate for that (as I found out many years later) by reading a great many manuscripts and, further, by writing to exhaustion. In particular, he would re-write the same text many times until it expressed, fully and properly, his ideas. No doubt his British and American colleagues and friends helped him with this task over the many years of his academic life.

My eight years in Sérgio’s lab were years of close scientific collaboration until I came to the Federal University of Minas Gerais, in 1985. Many students have followed in the Sergettes’ steps, and now it would be impossible for me to remember all those who were supervised by Sérgio Ferreira during their undergraduate course, as MSc and PhD students and as post-docs over the last thirty years. In truth, he always loved to have students around but overall he was enthusiastic and untiring in his search for new knowledge. He was very productive in his career, having written more than three hundred published articles, books and book chapters. I am certain that his example as a scientist has sustained not only my scientific life, but also that of all his students who had the privilege to share his professional life.

**Figure:**
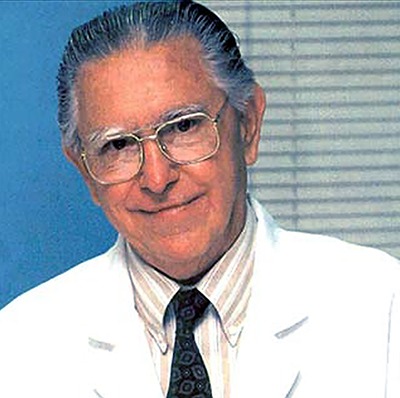


**Eduardo Moacyr Krieger**

## A remarkable career

*By Protásio L. da Luz, Instituto do Coração, Disciplina de Cardiologia, Faculdade de Medicina, Universidade de São Paulo, São Paulo, SP, Brasil*. protasio.luz@incor.usp.br

“There are men who fight one day and are good; others fight one year and they’re better, and there are those who fight many years and are very good; but there are the ones who fight their whole lives and those are indispensable”. Bertold Brecht.

It is a great pleasure and honor to write an appraisal of Eduardo Moacyr Krieger's (f3) career in this issue of the Brazilian Journal of Medical and Biological Research, which honors him as one of its founding fathers.

Eduardo Moacyr Krieger is a unique figure in Brazilian science. As in Bertold Brecht's words, he is indispensable. A scientist with an irreproachable medical and academic formation, he was born June 27, 1928 in Cerro Largo, RS. He obtained his medical degree from the Faculty of Medicine, Federal University of Rio Grande do Sul, in 1953. There he was introduced to the basic concepts of Medicine. He was influenced especially by his mentor: Rubens Maciel, a scholar of rare intelligence who personified competence and ethics. His post-graduate training occurred in Buenos Aires where he worked under Braun Menéndez and Bernardo Houssay. This team of investigators represented the pinnacle of science in South America at the time: Bernardo Houssay won the Nobel Prize of Medicine in 1947 and Braun Menéndez was one of the discoverers of angiotensin in 1940. Luis Leloir, who also received a Nobel Prize in chemistry in 1970, belonged to that team as well.

It was in Buenos Aires that Krieger's talent for research blossomed. He was introduced to the wonders, intricacies and challenges of new discoveries with their repeated sequences of failures and errors. He soon realized that the path toward new discoveries was not always smooth and that persistence is an essential component of achievements.

He then went to Georgia University, USA, where he was introduced to more sophisticated methods and crystalized his training in research. He worked under William Hamilton and met Raymond Ahlquist, the man who discovered the alpha and beta adrenoceptors. That was indeed a fantastic learning environment. Georgia University was a leading institution in hypertension research. Finally, rather than coming back to his native Rio Grande do Sul, he was attracted to the recently inaugurated campus of the University of São Paulo (USP) - the Faculty of Medicine of Ribeirão Preto. In 1957, he joined a renowned international team of investigators that would be companions for 20 years. Among those were Mauricio Rocha e Silva, Miguel Covian, Sérgio Ferreira and Lewis Greene. Then, upon retiring from his official academic responsibilities in Ribeirão Preto, he accepted an invitation from Prof. Fúlvio Pillegi, the Director of the Instituto do Coração - InCor, USP, and joined the Experimental Division of that institution in 1985, where he set up the Hypertension Laboratory. He has been working at InCor until today where he has served in several positions, exerting scientific, administrative and educational roles.

Thanks to his specialized training in cardiovascular physiology in Buenos Aires and the USA, Eduardo Krieger assembled extensive knowledge in this field. In addition, he worked in physiology and taught the subject for many years in Ribeirão Preto. The result of such long experience is a profound knowledge of cardiovascular physiology based especially on experimental studies. At InCor, he seized the opportunity to implement human studies as well. Therefore, he is one of the pioneers in translational medicine in the country. However, besides research, he dedicated himself to teaching the scientific method to post-graduate students of the InCor program. He was co-chair of a Course on Scientific Methodology for several years. His reputation as an authority on cardiovascular physiology is recognized worldwide. Testimony of such prestige is the fact that he is a full member of many scientific societies in Brazil and other countries as well. His contributions to national and international scientific organizations are remarkable.

In fact, he presided the Federação das Sociedades da Biologia Experimental (FESBE) for three terms (1985-91), the Academia Brasileira de Ciências (ABC) for two terms (1993-2007) and the Board of Administration of the Centro de Gestão e Estudos Estratégicos do Ministério de Ciência e Tecnologia (2002- present). Presently, he is a Member of the Superior Council and Vice President of Fundação de Amparo è Pesquisa do Estado de São Paulo (FAPESP), member of the Academia Nacional de Medicina, Co-Chair of the Executive Committee of the Inter-Academy Panel, which represents the conglomerate of Academies of Sciences of the world. He is also a co-founder of Sociedade Brasileira de Hipertensão (SBH) and its first President. More recently, he participated in the founding of the Associação Brasileira de Cardiologia Translacional (ABCT) of which he became Vice-President. In addition, he is an active member of several private foundations that support scientific and educational activities.

Presently, he is the Co-chair of the Internationalization Program of FMUSP. In this position, he contributed to the establishment of broad agreements with many universities throughout Europe, USA, Canada and Asia. These agreements presuppose bilateral exchanges of students and educational experiences in general. Among these are the University of Michigan (USA), Kyoto University (JAPAN) and Charité University (Germany).

As a member of the Board of Third World Academy of Sciences, he has promoted international meetings and debated innumerable problems related to technological innovation and scientific development, including climate, global environment, the relationship between governments and scientific communities, scientific and medical education. He received more than 20 scientific awards from national and international institutions including the Lifetime Achievement Award from the Inter American Society of Hypertension and the Mühlbock Award, from the International Council for Laboratory Animal Sciences (ICLAS). In particular, he received the Scientific Merit Award, category Gran Cruz, of the Ministry of Science and Technology, the highest scientific distinction from the Brazilian government. He is also an Emeritus Professor of the Faculty of Medicine, Ribeirão Preto.

It can be safely said that science in general, technological development, and education have been life-long preoccupations of Eduardo Moacyr Krieger. To these issues, he has dedicated a major part of his time and indeed contributed immensely to their advancement. In all these positions, he proved to be an efficient organizer, a visionary and a devoted leader with a universal vision of the scientific mission of those institutions.

## Integrity

A critical characteristic of a scientist is integrity. In science, it is not possible to check *in loco* every experiment or scrutinize to whom a primary idea or concept belongs. Hence, to a large extent science deals with searching for and reporting the truth incessantly. Reliability, therefore, is its essence. A real scientist must refrain from exaggerating the importance of his own contributions but, he also must recognize the merits of fellow investigators. Furthermore, he needs to be aware of limitations of the scientific methods, even the most sophisticated ones, so as not to trust excessively primary discoveries, even when they seem exciting and revolutionary. In other words, he must refrain from adapting data to his own pre-conceived ideas. He must stay away from “wishful thinking”. In short, a scientist is a slave of the truth, although absolute truth is often elusive. Also, in the present era when commercial interests permeate medicine and research so frequently, the real scientist must keep a safe distance from those interests.

For Eduardo M. Krieger, it can be said, undoubtedly, that he never bowed to any of those temptations. He is a living example of scientific integrity.

## Work Capacity

Those who are close to him are always amazed by his ability to work long hours, everyday. Also impressive is his ability to deal with different subjects at the same time. That requires tremendous mental discipline that certainly is inherent to his personality but is also the result of constant practice. His capacity for concentration is absolutely uncommon. He does not waste time except for sipping a good wine with friends!

## Coherence

Eduardo M. Krieger chose the path to his professional career from his early days as a student and young investigator: he is essentially a scientist and an educator. He worked incessantly for the progress of science in his country and over his long career never missed this objective. As other great educators and scientists, he never confused his mission. He knew perfectly that his mission in life was to pursue scientific truth, to advance knowledge through his own research and to disseminate that knowledge among colleagues, but especially, among his trainees.

Thus, a major focus of Eduardo M. Krieger has been education. To educate, one must dominate a given subject, from its basic concepts to practical applicability and future perspectives, and possess the patience, the determination and the ability to transmit knowledge. Knowledge can be transmitted both by practical performance, as in experimental laboratories, but also by oral or written concepts. Clarity is essential; this requires an organized, methodic mind. Besides, teaching requires understanding other people’s mind and souls, so that lessons can be individualized. A mentor, in certain ways, is like a psychologist, who can identify sentiments, interpret them and even direct aspirations. Understanding one’s personality is a key element to successful mentorship. Just as everyone is unique and can sense facts in very personal ways, learning is also a very personal process. Thus, respecting those individualities is essential to successful teaching. Furthermore, a mentor needs to enjoy teaching, and take pride in their student’s accomplishments; but above all, a mentor shall give examples of dedication, professional excellence, persistence, discipline, and accomplishment. Most of all, he must inspire his trainees, identify talents, and promote their intellectual growth.

Eduardo M. Krieger has it all. He formally trained more than 30 PhD students, many of whom are academic professors and created their own research laboratories, as mentioned by Vasquez (1). This is testimony to his permanent dedication to education and demonstrates the profound influence of Eduardo M. Krieger upon the formation of young scientists in Brazil.

## Scientific Contribution

He studied, specifically, regulation of arterial pressure by the peripheral and central nervous systems. He observed in rats that stimulation of the vagus nerve caused oscillations in blood pressure. He created the sino-aortic denervation (SAD) model, which has been used by many laboratories and is cited extensively (2). He gave special attention to baroreflex control of blood pressure and demonstrated resetting of baroreflexes in conditions of sustained hypertension in rats. These studies resulted in several publications (3-8) and represent original and fundamental contributions to the understanding of mechanisms underlying hypertension. More recently, he focused on human resistant hypertension, studying specifically the effects of aldosterone and clortalidone. He chaired the Resistant Hypertension Optimal Treatment Trial (9 [Bibr B10] [Bibr B11] [Bibr B12] [Bibr B13] [Bibr B14] [Bibr B15] [Bibr B16] [Bibr B17] [Bibr B18]) in Brazil whose results will be published soon. Preliminary data of 2000 patients indicate that the prevalence of resistant hypertension is about 14% and that both spironolactone and clonidine are effective treatment options.

## Human Relationships

He has been married to Lorena Catarina Krieger and has a son, José Eduardo Krieger, who is Full Professor of Genetics at the Faculty of Medicine, USP. His daughter Marta Helena is a past Professor of Biology at UNICAMP. He has three grand-children. He is a family man who found in his family constant and warm support.

Krieger is a true gentleman. With affable manners, he treats everyone with respect. Among colleagues, he is highly respected precisely because he is always willing to help and is trustful; in sum, he is an honorable man who keeps his word. One of his traits is that he constantly thinks of broad ideas and policies that can help the community. He is a humble man who never puts himself ahead of anybody. He is at the same time a visionary who dreams of a better future for his country and a conciliatory person who believes in understanding and comprehension. He believes more in common efforts and cooperation rather than diversion. He looks for and, hence, finds more positive qualities than defects in others. Vision, comprehension, optimism, determination, and constant work made him one of the most important leaders of science in Brazil.
